# Reference Hallucination Score for Medical Artificial Intelligence Chatbots: Development and Usability Study

**DOI:** 10.2196/54345

**Published:** 2024-07-31

**Authors:** Fadi Aljamaan, Mohamad-Hani Temsah, Ibraheem Altamimi, Ayman Al-Eyadhy, Amr Jamal, Khalid Alhasan, Tamer A Mesallam, Mohamed Farahat, Khalid H Malki

**Affiliations:** 1 College of Medicine King Saud University Riyadh Saudi Arabia; 2 Department of Otolaryngology, College of Medicine Research Chair of Voice, Swallowing, and Communication Disorders King Saud University Riyadh Saudi Arabia

**Keywords:** artificial intelligence (AI) chatbots, reference hallucination, bibliographic verification, ChatGPT, Perplexity, SciSpace, Elicit, Bing

## Abstract

**Background:**

Artificial intelligence (AI) chatbots have recently gained use in medical practice by health care practitioners. Interestingly, the output of these AI chatbots was found to have varying degrees of hallucination in content and references. Such hallucinations generate doubts about their output and their implementation.

**Objective:**

The aim of our study was to propose a reference hallucination score (RHS) to evaluate the authenticity of AI chatbots’ citations.

**Methods:**

Six AI chatbots were challenged with the same 10 medical prompts, requesting 10 references per prompt. The RHS is composed of 6 bibliographic items and the reference’s relevance to prompts’ keywords. RHS was calculated for each reference, prompt, and type of prompt (basic vs complex). The average RHS was calculated for each AI chatbot and compared across the different types of prompts and AI chatbots.

**Results:**

Bard failed to generate any references. ChatGPT 3.5 and Bing generated the highest RHS (score=11), while Elicit and SciSpace generated the lowest RHS (score=1), and Perplexity generated a middle RHS (score=7). The highest degree of hallucination was observed for reference relevancy to the prompt keywords (308/500, 61.6%), while the lowest was for reference titles (169/500, 33.8%). ChatGPT and Bing had comparable RHS (β coefficient=–0.069; *P*=.32), while Perplexity had significantly lower RHS than ChatGPT (β coefficient=–0.345; *P*<.001). AI chatbots generally had significantly higher RHS when prompted with scenarios or complex format prompts (β coefficient=0.486; *P*<.001).

**Conclusions:**

The variation in RHS underscores the necessity for a robust reference evaluation tool to improve the authenticity of AI chatbots. Further, the variations highlight the importance of verifying their output and citations. Elicit and SciSpace had negligible hallucination, while ChatGPT and Bing had critical hallucination levels. The proposed AI chatbots’ RHS could contribute to ongoing efforts to enhance AI’s general reliability in medical research.

## Introduction

Artificial intelligence (AI) evolved from the early twentieth century until Turing [1] conceptualized machine learning in the 1950s and introduced the idea of using machines to process information in order to solve problems and make decisions. Since then, scientists and others have pursued the dream of creating machines that mimic human intelligence through cognitive processes such as thinking, data processing, and planning. AI development requires foundational work in spoken language processing and information storage. Achieving these milestones involves numerous obstacles, including significant technical and financial challenges, which delay the maturation of AI [2].

A major hurdle in AI development was natural language processing, which progressed through multiple phases and culminated with the invention of bidirectional encoder representations from transformers, self-attention, and sequence-to-sequence deep learning technologies. These transformers marked a pivotal advancement in AI development, generating fluent and coherent large language models (LLMs), as they enabled the analysis of each word in an input sequence in context to its neighbors on both sides [3].

In 2018, OpenAI unveiled its first Generative Pre-trained Transformer, ChatGPT, enhancing its utility and potential applications in various human activities, including health care and medical research sectors. Initially, ChatGPT stored data in its database without access to continually updated human literature. It later evolved to employ internet access. Subsequently, other AI chatbots specializing in health care and medical research were introduced. The implementation of AI chatbots in health care is optimistically viewed as a means to improve health care systems, medical education, and patient outcomes [4-6]. They are also viewed as potentially valuable tools in medical research and manuscript writing due to their capabilities in organization, data processing, text generation, and summarization [7,8].

Since the public launch of ChatGPT at the end of 2022, the medical literature surrounding AI has expanded tremendously [9]. Most studies have addressed its potential contributions to medical research and health care practices. However, a critical analysis of the literature reveals that a minority of AI chatbot users realized early that these LLMs do not voluntarily cite credible references to authenticate their information [10-12]. Even when ChatGPT and other AI chatbots were challenged to authenticate their outputs with references [13,14], they generated multiple citations with detailed bibliographic data that seemed perfectly authentic, but most were actually fabricated, contained at least one falsified citation data, or were completely erroneous when verified through medical literature resources [13-16].

These fictitious citations raised major concerns about the AI chatbots’ algorithms and methodology of natural language processing, especially regarding references, their bibliographic data credibility, and authenticity. Such erroneous outputs were described in the literature as AI hallucinations or fabrications. The mechanism of these fabrications remains unclear, though some AI developers have identified this faulty output and described it as a misalignment between user expectations and AI chatbot capabilities related to machine learning training issues without proposing definite explanation for that phenomenon or a methodology to assess its references’ hallucination degree or impact on AI platform output [17]. Another possible explanation for reference hallucinations is an encoding/decoding transformer glitch or the way AI chatbots handle bibliographic data as text amenable to manipulation. Some investigators assessed the AI-generated medical information, including the ability to verify references and its publication date, but did not pay attention specifically to certain identifiers of references that help to verify their authenticity [18].

Investigating the sources of these hallucinations and taking serious steps to fix the contributing factors to this phenomenon is an urgent need in the current stage to implement AI technology in health care practice and medical research with high credibility. The first step is to identify the degree of these hallucinations, especially in relation to references and their bibliographic data across different AI chatbots.

Utilization of AI-generated medical information is becoming a routine tool for physicians and trainees in medical education and even for diagnosis and management. Therefore, the verification of references for this information is extremely important to avoid adopting or utilizing erroneous unverified information and might be disastrous if used in medical management. Therefore, we proposed to construct a reference evaluation tool that can be universally applied to assess AI chatbot–generated references, which would be a helpful tool for assessing the output, its credibility, and its ability to be implemented into practice. This study aims to introduce this tool as Reference Hallucination Score (RHS) and to demonstrate its application in evaluating and comparing the outputs of 6 AI chatbots, stratify their citation output according to the RHS, and assess the variables that are associated with RHS. This initiative is the first to specifically address the gap in evaluating AI referencing hallucinations, thereby offering a unique approach that fills a critical gap in enhancing the reliability of AI-generated references, improving trust in AI outputs within medical research, and guiding the development of more robust and reliable AI models.

## Methods

### Medical Prompts

We challenged 6 AI chatbots with 10 medical prompts addressing various medical topics. The selected AI chatbots were ChatGPT 3.5, Bard, Perplexity, Bing, Elicit, and SciSpace [19-24]. We chose them because these are the most widely used AI chatbots in the literature and medical research and because of their ease of access by users. Using the focus group technique, we structured 10 medical prompts with the best possible textual format and clarity to be understood by the AI chatbots. Each of the 2 prompts addressed a similar medical topic: the first in a basic format and the second in a complex or scenario-based format. The prompts are provided in Multimedia Appendix 1. The 5 general topics for the medical prompts were glucose control in gestational diabetes, triggering factors in older adults with asthma, septic shock in infants with severe combined immunodeficiency, arthritis in patients with sickle cell disease, and substance abuse in patients with personality disorders.

All the prompts were formulated according to the following format: (1) begin by searching PubMed for papers related to the topic; (2) review the search results and select 10 relevant and recent papers; (3) ensure that all information is accurate and up-to-date; (4) format the list of papers in a clear and organized manner, using a consistent style for each entry; (5) include any additional information or notes that may be relevant or helpful for readers; and (6) double-check the accuracy and completeness of the list before publishing or submitting it. The same 10 prompts were applied to each of the 6 AI chatbots. Each prompt requested the AI chatbots to compile a list of 10 references. Each prompt listed 7 items for every reference: reference title, journal name, authors, DOI (digital object identifier), publication date, paper web link, and the reference’s relevance to the keyword prompts.

### RHS Development

Using the Delphi technique, we proposed the RHS. Score development went through a robust and meticulous methodology as follows:

#### Literature Review

We commenced with an exhaustive review of existing literature to identify unique identifiers for reference scoring. This step was vital for understanding the current landscape and ensuring that our tool addresses the new gap in the field.

#### Expert Consultation Using the Delphi Technique

Our approach utilized the Delphi technique—a systematic, multistep process involving rounds of anonymous feedback from experts to reach a consensus. We detail the implementation of this technique as follows.

1. Initial survey of senior librarians: We consulted 2 senior librarians to gather insights on the proposed bibliographic identifiers, seeking their expert suggestions for improvements.
2. Expanded survey of the senior physicians: We further extended our consultation to 12 senior physicians and 11 junior physicians, most of whom are academicians, to assess the relevance and importance of the suggested bibliographic identifiers and to collect additional suggestions.
3. Consensus building among authors: The final step involved synthesizing the feedback received and reaching a consensus among the authors, based on the mean results from the surveyed academics, to finalize the bibliographic identifiers for the hallucination score.

The RHS is an AI chatbot scoring system to evaluate paper references generated by the AI chatbot based on the hallucination severity. The RHS is calculated based on the total score according to the presence of hallucination in any of the 7 parameters (Table 1). The parameters are 4 reference items or identifiers given a score of 2 if they encountered any error in the reference title, journal name, authors’ names, or DOI, as the authors judged it as a major degree of hallucination. A score of 1 was given to any error in any of the other 3 identifiers, that is, reference publication date, reference web link, or reference relevance to the keyword prompts, as they were judged as minor degrees of hallucination. The maximum RHS is 11, indicating the maximum degree of reference hallucination, and the minimum RHS is 0, denoting no reference hallucination. To achieve the best outcome from the proposed RHS, the prompts submitted to the AI chatbot should include clear instructions to include all 7 referencing items. The authenticity of the citations’ items is evaluated by comparing AI chatbot responses to PubMed and Google Scholar responses. If the AI chatbot could not produce any reference to a specific prompt, the AI chatbot was given a score “N” for that prompt and was scored as failing to generate a result.

**Table 1:** Reference hallucination score (total score=11).

Reference identifier	Item hallucination score
Erroneous date of publication^a^	1
Erroneous web link of the paper^b^	1
Erroneous citation relevance^c^	1
Erroneous title of the paper^d^	2
Erroneous digital object identifier^e^	2
Erroneous authors’ names^f^	2
Erroneous name of the journal^g^	2

^a^The publication date is missing or inaccurate.

^b^The link to the paper is missing or directs to a different paper or an error page.

^c^The keyword prompts are not in the title, abstract, or reference keywords.

^d^The title provided by the artificial intelligence platform is misspelled, incomplete, or nonexistent.

^e^Digital object identifier is missing, inaccurate, nonexistent, or directs to a different paper.

^f^Any author’s name is missing, misspelled, or nonexistent.

^g^The journal’s name is missing, misspelled, did not publish the paper, or does not exist.

### Testing AI Chatbots

Our methodology of judging the hallucinations depended on using a systematic verification process based on PubMed and Google Scholar to ensure legitimacy, accuracy, and transparency. Each reference was initially verified using PubMed by searching for its DOI. If the DOI was inconclusive or unavailable, we leveraged a combination of the paper’s title, author names, and other vital details to ascertain its existence and accuracy within the PubMed database. In case of failure to reach the reference through PubMed, Google Scholar was the second step for verification using DOI, the paper’s title, authors, and other pertinent details to ensure the reference authenticity.

Each reference’s total hallucination score was calculated according to the RHS methodology. Then, the mean RHS of the produced references of each prompt was calculated. This was applied to the 6 AI chatbots. The chatbots’ RHS items were compared across the studied chatbots according to hallucination, correct results, and failure to generate results. The median RHS of the complex prompts was compared with basic prompts, and finally, the median RHS of each AI chatbot was compared across all the studied chatbots. Linear regression was used to assess the independent association between mean RHS and other predictors, namely, the studied chatbot, prompt type, and prompt iteration.

### Statistical Analysis

The mean (SD) values were used to describe continuous variables. The median (IQR) values were used to describe continuous variables with statistical evidence of skewness. The frequencies and percentages were used to describe categorically measured variables. The Kolmogorov-Smirnov statistical test and histograms were used to assess the statistical normality assumption of the metric variables. The categorical Cronbach α test was used to assess the internal consistency for the reliability of the 7 parameters of the RHS. The nonparametric Kruskal-Wallis analysis of variance test was used to compare the RHS item results across the studied chatbots and to compare the median RHS for statistically significant differences. The Mann-Whitney *U* nonparametric test compared the chatbots’ median RHS for statistically significant differences between basic and advanced prompts. The multivariable generalized linear models with gamma regression analysis assessed significant differences in the mean RHS by regressing it against the AI chatbot, prompt complexity, and prompt iterations. The association between the mean RHS and tested predictor independent variables was expressed as an exponentiated β coefficient (risk rate) with its associated 95% CI. The SPSS statistical computing software (IBM Corp) was used for the statistical data analysis, and the α significance level was considered at .05.

### Ethical Considerations

This paper did not involve research on living creatures; therefore, no institutional review board approval was required.

## Results

A total of 10 prompts were entered into each AI chatbot. Half inquired about the basic medical topics, and the other half about clinical scenarios or complex medical topics. Each prompt requested 10 references related to the prompt topic with their reference data according to our research methodology. Bard was the only AI chatbot that failed to produce any response to all the 10 applied medical prompts. Bard’s response to our prompts was, “I’m a language model and don’t have the capacity to understand and respond.” The AI chatbots failed to generate any reference response for 35 (7%) of the 500 references. The highest hallucination/erroneous output was for the reference relevancy to the prompt content (308/500, 61.6%), followed by publication date (237/500, 47.4%), authors’ names (228/500, 45.6%), DOI (227/500, 45.4%), and reference web link (187/500, 37.4%). Regarding the reference title and journal name, the AI chatbots’ output had hallucination results of 33.8% (169/500) and 37.6% (188/500), respectively.

Figure S1 in Multimedia Appendix 1 shows each AI chatbot’s reference results (correct and hallucinating or erroneous results). SciSpace and Elicit had the highest correct reference identifiers’ results, with 629 and 597, respectively. ChatGPT had the highest hallucination results at 592. Bing had the highest rate of failure to generate results at 210. The Kruskal-Wallis nonparametric test showed that the AI chatbots differed significantly with respect to their total hallucination results (*χ*^2^_4_=205.9; *P*<.001), correct results (*χ*^2^_4_=305.0; *P*<.001), and failure to generate results (*χ*^2^_4_=104.3; *P*<.001). The Bonferroni-adjusted post hoc pairwise comparison test was used to compare the chatbots. SciSpace and Elicit did not differ significantly with respect to their hallucination or correct results (*P*>.99). Both had significantly fewer hallucinations and higher correct results compared to the other chatbots (*P*<.001). ChatGPT had the highest rate of hallucination results. Bing and ChatGPT had no significant difference in their correct results (*P*>.99). Bing and Perplexity had no significant difference in their hallucination results (*P*>.99) and were significantly superior to ChatGPT. Perplexity was superior to both regarding its correct results (Bing, *P*=.002; ChatGPT, *P*=.004). Bing had the highest rate of failing to generate results compared to the rest (*P*<.001), followed by Perplexity.

Table 2 displays the detailed results of the studied AI chatbots’ reference characteristics. The Kruskal-Wallis nonparametric test showed that the AI chatbots differed significantly in their results. SciSpace and Elicit generated the lowest number of hallucination results regarding title, journal name, authors’ names, DOI, and reference web links compared to all the other AI chatbots (*P*<.001). However, Elicit and Perplexity showed no significant difference in hallucinating the publication date results, and both hallucinated more than SciSpace.

**Table 2:** Artificial intelligence chatbot hallucinating/erroneous reference identifiers’ results.

Reference identifier	ChatGPT	Perplexity	SciSpace	Elicit	Bing	Chi-square *(df)*	*P* value
Title	82	41	0	0	46	231.4 (4)	<.001
Digit Object Identifier	89	73	8	4	53	261.26 (4)	<.001
Journal name	83	56	0	0	49	249.4 (4)	<.001
Authors’ names	89	74	0	0	65	341.1 (4)	<.001
Publication date	88	48	0	40	61	199.9 (4)	<.001
Reference web link	84	49	3	1	50	231.4 (4)	<.001
Reference relevance to topic prompt	77	63	60	58	50	10.77 (4)	.03

Perplexity, Bing, and ChatGPT had no significant difference regarding their hallucination results for DOI, authors’ names, reference web links, and reference titles. Perplexity and Bing hallucinated significantly lesser than ChatGPT regarding the journal name. Perplexity hallucinated slightly lesser than ChatGPT regarding reference title. Bing had significantly fewer hallucination results for references’ irrelevancy to the prompt medical topic than ChatGPT (*P*=.045). The remaining AI chatbots, including Bing, did not show a significant difference (*P*>.05).

Table 3 shows AI chatbots’ RHS with the descriptive analysis of the median (IQR) for each studied AI chatbot. Kruskal-Wallis analysis showed that chatbots differed significantly with respect to their overall measured hallucination scores (*χ*^2^_4_=277.7; *P*<.001). ChatGPT and Bing had the highest median RHS but did not differ significantly (*P*>.99). SciSpace and Elicit had the lowest median RHS (*P*<.001) and did not differ significantly (*P*>.99). Perplexity and Bing did not vary significantly with respect to their median total RHS (*P*=.19). On the other hand, Perplexity had a considerably lower median total RHS than ChatGPT (*P*=.003). For the prompt complexity, the median RHS did not differ significantly for each of the studied AI chatbots (*P*>.05).

**Table 3:** Reference hallucination scores with bivariate analysis of the artificial intelligence chatbots.

Chatbot	Hallucination score, median (IQR)^a^				*z* test statistic *(df)*		*P* value
	Total RHS^a^	Basic prompts RHS	Advanced prompts RHS				
ChatGPT	11 (1)	11 (1)	11 (0.25)	1.25 (100)		.21	
Perplexity	7 (5)	7 (8.25)	8 (5)	0.837 (95)		.40	
SciSpace	1 (1)	1 (1)	1 (1)	0.942 (100)		.35	
Elicit	1 (2)	1 (2)	1 (2)	0.207 (100)		.84	
Bing	11 (6)	11 (6)	11 (5)	0.207 (70)		.84	

^a^RHS: reference hallucination score.

Table 4 shows the multivariable generalized linear models with γ regression of the mean total hallucination score based on the chatbot, prompt complexity level, and prompt type. ChatGPT and Bing had the highest mean total hallucination scores and did not differ significantly (*P*=.32). SciSpace had the lowest mean total hallucination score compared to ChatGPT (β coefficient=–1.748; **P*<.001*). Elicit had the second lowest total mean hallucination score compared to ChatGPT (β coefficient=–1.63; *P*<.001). Perplexity had the third lowest score compared to ChatGPT (β coefficient=–0.345; *P*<.001).

**Table 4:** Multivariable generalized linear mixed regression analysis of the artificial intelligence chatbots’ total hallucination score^a^.

	β coefficient (95% CI)	*P* value
Intercept	2.142 (1.997 to 2.288)	<.001
Chatbot vs Bing	–0.069 (–0.206 to 0.067)	.32
Chatbot vs Elicit	–1.630 (–1.769 to –1.492)	<.001
Chatbot vs SciSpace	–1.748 (–1.880 to –1.617)	<.001
Chatbot vs Perplexity	–0.345 (–0.510 to –0.181)	<.001
Prompt complexity vs advanced level	0.486 (0.326 to 0.645)	<.001
Prompt number	0.018 (–0.006 to 0.041)	.10
Interaction effect: prompt number vs prompt complexity	–0.077 (–0.108 to –0.046)	<.001

^a^Dependent outcome variable: reference hallucination score+1; probability distribution = gamma link function with log shape.

The level of prompt complexity also significantly affected the hallucination score when compared across all the AI chatbots. The advanced prompts’ mean total hallucination score was significantly higher than the basic prompts’ mean total hallucination score (β coefficient=0.486; *P*<.001). The prompt topic did not correlate significantly with the AI chatbots’ mean hallucination score (*P*=.14). However, the interaction term between the prompt medical topic and prompt complexity was found to be statistically significant (β coefficient=–0.077; *P*<.001), indicating that some topics, when presented to the chatbots in a complex scenario, resulted in significantly lower mean total hallucinations compared to the basic presentation of the same topic (*P*<.001). The mean hallucination score based on prompt topic and prompt complexity did not significantly interact with any specific AI chatbots studied.

## Discussion

### Principal Findings

Our findings showed variations in the RHS across different AI chatbots, ranging from almost null for SciSpace and Elicit to a critically high degree of hallucination for ChatGPT [25]. Among the bibliographic items we studied, the publication date (237/500, 47.4%) showed erroneous or hallucinating results, while the reference title (169/500, 33.8%) showed the least hallucinating or erroneous results. Reference relevancy to the prompt topic was the most common source of hallucination, ranging from 50 erroneous results in case of Bing chatbot up to 77 erroneous results in case of ChatGPT. Bard failed to generate any references for all the studied 10 prompts.

The scientific community uses a transparent, reproducible, and accessible archiving system for its large, evolving research data. For example, FAIR (Findability, Accessibility, Interoperability, and Reuse) guidelines ensure that reference data are archived in a findable, accessible, interoperable, and reusable format to facilitate its citation, maintain researchers’ credibility, and ensure data integrity and nonrepudiation [26]. Citation is a vital step for information verification and authentication. AI chatbots that have gained recent widespread use still lack a transparent and robust system for verifying citations and their information sources. Additionally, AI chatbots encounter a hallucination/fabrication phenomenon recognized early in their use in health care. Hallucinations have been encountered in various domains, including the content itself and the cited references, including their bibliographic identifiers [12,27-29]. However, it is worth noting that this obstacle is improving gradually, especially by introducing research, dedicated medical chatbots, and upgrading existing ones [16,30].

A possible explanation for AI chatbots’ referencing hallucination is the methodology LLMs use to handle citations. LLMs may deal with citations and their bibliographic identifiers as text, making them vulnerable to paraphrasing and other linguistic manipulations and perhaps as a watermark so that the output that is generated by AI could be identified versus that produced by humans to reduce AI misuse [29,31,32]. Buholayka et al [33] reported that ChatGPT is trained to give uninterrupted flow of conversation even at the cost of giving hallucinating results. Another possible mechanism related to AI chatbots’ natural language processing methodology involves encoding and decoding defects during prompt processing, generating errors, and fabricating results [34]. Additional factors might include insufficient training data for the LLM [17,35,36], context misinterpretation, lack of external validation, and overreliance on pattern recognition, all of which could contribute to variable degrees of hallucination, depending on the specific LLM and prompt structure.

Ye et al [37] constructed a systematic approach to AI chatbot hallucination by introducing a unique classification of hallucinations across diverse text generation actions, thereby furnishing theoretical insights, identification techniques, and enhancement strategies. Their methodology consists of 3 domains: (1) comprehensive classification for hallucinations manifested in text-generation tasks, (2) theoretical examinations of hallucinations in LLMs and amelioration, and (3) several research trajectories that hold promise for future exploration. Dhuliawala et al [38] suggested another model to potentially reduce hallucination by the chain-of-verification method. Their 4-step process consists of the chatbot drafting its initial response, formulating verification questions to scrutinize the draft, addressing these questions independently to prevent bias, and finally, generating a thoroughly verified response. Further research needs to be performed to see which model and which LLM receives a better hallucination score with time.

Our findings align with those of Hua et al [25] who evaluated hallucinations in AI-generated ophthalmic scientific abstracts and references. The uniqueness of our pioneering work is the introduction of a novel RHS to assess AI chatbots. RHS is based on 6 important reference items that make each reference unique and easily trackable for citation, in addition to a seventh item related to the relevance of the reference to the topic prompt [26]. The score was constructed based on any reference’s most usable and unique items, with differential weights according to their uniqueness and importance in tracking and identifying any reference.

Variations in hallucination degrees among the different bibliographic items highlight the variations among AI chatbots in handling and identifying references. Having a critically high hallucination rate of the cited reference’s relevance to the prompted topic stresses the possibility that AI chatbots identify certain keywords in the prompt and try to search for relevant references but, most of the time, fail to identify the correct and relevant keywords, or as described by a recent OpenAI company statement, they do not align properly with user intentions, leading to the citation of relatively irrelevant sources [17]. Further, hallucinating the paper title and DOI is risky in terms of inability to access the reference. Other identifier hallucinations such as journal name or authors may not greatly block the access to the cited reference.

According to our study, ChatGPT 3.5 had the highest total hallucination score, with the most hallucinations in all aspects of bibliographic items. This echoes other observations. Walters and Wilder [16] described many hallucinations in ChatGPT’s bibliographic citations. Their study used ChatGPT 3.5 and ChatGPT 4 to generate literature reviews. They analyzed 636 bibliographic citations across 84 papers, finding a significant number of fabricated citations (55% for GPT 3.5, 18% for GPT 4) and errors in the nonfabricated ones (43% for GPT 3.5 and 24% for GPT 4). Despite GPT 4 showing notable enhancement and insights over GPT 3.5, fabricated references persist [39]. Bing’s total hallucination score did not differ significantly from that of ChatGPT as they both had critically high scores. SciSpace and Elicit had comparable individual rates of hallucination regarding the different bibliographic items, although SciSpace had the lowest total number of hallucinations, followed by Elicit. When considering the different studied reference items, ChatGPT, Bing, and Perplexity had comparable hallucinating results apart from the journal name, where Bing and Perplexity hallucinated lesser than ChatGPT. Perplexity stood in the middle, as its overall hallucination score was worse than SciSpace and Elicit on one side and better than ChatGPT and Bing on the other. Our observations align with those of others who investigated AI platforms for writing and research objectives, as they found that Elicit and SciSpace are far more dedicated to searching for scientific papers and summarizing references [40]. This observation stresses scholars’ urge to vigilantly examine reference accuracy for any LLM-generated citations, especially if they are not dedicated to research purposes [12].

An interesting observation from our study is the failure of some chatbots to generate the prompted citations, as ChatGPT, Bing, and Perplexity had comparable individual hallucination reference results apart from journal names, where Bing and Perplexity hallucinated lesser than ChatGPT. Bing had a significantly higher rate of failure than ChatGPT, even though it performed comparably in all aspects. Such performance by Bing has been observed in another study that prompted ChatGPT, Bard, and Bing for multiple choice question generation, where Bing had a significant rate of generation failure compared to the other two [41].

Prompt structure and complexity had an interesting association with hallucination score, as complex or clinical scenarios triggered significantly more hallucinations across AI chatbots but not for certain ones. This was also observed when challenging ChatGPT versions 3.5 and 4 with orthopedic questions matched with images, as they both performed far better with simple text multiple choice questions than those with images [42]. On the other hand, when prompted in a complex or scenario format, specific medical topics caused lesser hallucination than when prompted in a basic format. This observation might be explained by the transformer’s methodology and their differential performance with different text structures.

Bard performance might point to serious glitches in its text recognition or transformer performance in medical research, at least in certain topics or in the ones used in our study. Previous work has shown serious fabrications encountered in Bard similar to ChatGPT [43]. Overall, our observations extend other findings that AI chatbots provided citations with varying degrees of inaccuracy or hallucination, necessitating users to independently verify the information obtained from these language models [43]. As such, hallucinations put the whole AI chatbot data output under significant question, especially in implementing and applying AI aid into clinical practice [44,45].

Our study incorporates a meticulous design by constructing a novel scoring system to assess AI chatbots’ hallucination in relation to references and their reference items with differential weights based on their importance. The multifaceted verification approach that we adopted is a robust method to ascertain the authenticity and relevance of the references generated, providing a replicable model for future studies. That score has proven to differentiate the performance of 5 common chatbots skeptically. The invention of a hallucination score will be a vital step toward systematically evaluating and improving the referencing capabilities of AI chatbots and LLMs. It will also triage AI chatbots’ hallucinations, which is a critical step in verifying the authenticity of their content.

### Study Limitations

Our study has potential limitations. The methodology used to construct the RHS is novel and is liable for future improvements. The RHS included a limited number of bibliographic parameters, which we believed, based on our consensus and expert colleagues of academics, are the most important and unique reference identifiers. However, other researchers might perceive additional variables or identifiers as crucial. The prompt structure that we used was after extensive trials to reach the prompt structure that produces AI-generated references and their identifiers accurately as much as possible. Still, the prompt design is liable for limitation, as LLM output depends hugely on the prompt structure fed to them. Prompt structure might explain partly the failure of Bard Chatbot to generate any references, as it might need a special design of prompting. However, we do not believe that the prompt strategy was a major limitation, as it succeeded in generating outputs in almost all the other studied AI chatbots. Additionally, the medical prompts utilized might have a limited scientific scope, which may not cover the full spectrum of medical topics and scenarios that LLMs might encounter in real-world applications. Yet, proposed prompts to assess AI chatbots’ referencing hallucination require future refinement, especially with the introduction of new AI chatbots and specifically those that are specialized in the medical field.

Regarding the verification process of the references’ identifiers, even though we utilized multiple web-based steps utilizing PubMed and Google Scholar, this might still be suboptimal because although we relied heavily on existing databases and search engines, those engines might have their own set of limitations in indexing or recognizing all published literature, and future researchers might propose more universal agreed-on methodology in that regard. We selected only 6 chatbots for assessment; this might not provide a fair and comprehensive understanding of the hallucination problems encountered across the myriad of AI chatbots that are increasingly becoming available and more specialized. The potential biases in selecting AI chatbots and medical prompts, along with the verification process used, might impact the study results by not fully representing the breadth of AI capabilities and challenges. These limitations should be addressed in future research by expanding the number of chatbots studied, diversifying the medical prompts and their structure addressing medical basic knowledge, diagnosis, and management, in order to strengthen the generalizability of the findings. Finally, the verification processes need to be refined. Our proposed RHS needs future sharpening and application to different and future AI chatbots, especially medical ones, to test its generalizability and sensitivity to assess reference hallucination. Adding more bibliographic parameters might enhance its sensitivity. Furthermore, refining the definition of erroneous citations could potentially improve RHS performance and applicability, particularly in relation to the relevance of the references to the prompted topic.

### Conclusion

Our novel RHS tool encompasses a methodology for delineating referencing inaccuracies, crucially in medical domains. It has shown variations across the analyzed AI chatbots. We evaluated the performance of 6 common AI chatbots. Elicit and SciSpace had the least hallucination, with almost none, while ChatGPT and Bing had a critical degree of hallucination. This emphasizes the pressing need for enhanced evaluation mechanisms of AI chatbots’ output, particularly the cited references, and highlights the need to verify their output and apply it skeptically, all in order to grade AI chatbots’ credibility in terms of their contribution in health care and medical research areas. Additionally, our work establishes a foundation for ensuing research aimed at augmenting the reliability of AI chatbots in academic and clinical landscapes. Improving the LLM mechanism of reference recognition and handling is an important necessity and needs maturation and improvement of the algorithms. Training in user prompt strategy is another trajectory to address to achieve the best performance of these chatbots and improve chatbot-user alignment. Future improvement of RHS or developing new versions will improve AI chatbot assessment and categorization and potentially help AI engineers to evaluate their work. The significance of RHS and its potential impact on improving the reliability of AI-generated references cannot be overstated. The key takeaways highlight the broader implications for the use of AI in medical research, emphasizing the necessity for rigorous evaluations to enhance trust and reliability in AI outputs.

## Appendix

Multimedia Appendix 1 Data of the medical prompts.

## Abbreviations

AI: artificial intelligence
DOI: digital object identifier
FAIR: Findability, Accessibility, Interoperability, and Reuse
LLM: large language model
RHS: reference hallucination score

## Data Availability

The data may be made available upon reasonable request to the corresponding author.
