# Supplementary material for: Reference Hallucination Score for Medical Artificial Intelligence Chatbots: Development and Usability Study
Source: JMIR Med Inform. 2024 Jul 31;12:e54345. doi: 10.2196/54345 (PMC11325115; doi:10.2196/54345)
Supplement: Multimedia Appendix 1 [file medinform_v12i1e54345_app1.docx]

## **Multimedia Appendix 1**

*Prompt 1 (Basic):*

Act as an experienced researcher. Compile a list of ten recent articles on the variables that affect glucose control in gestational diabetes.

Include the following information for each article:

1. Article title.

2. Author(s).

3. Journal name.

4. Date of publication.

5. Number of citations.

6. DOI.

7. Web link to the article.

8. PubMed link.

Instructions:

1. Begin by searching PubMed.

2. Review the search results and select ten recent articles that are relevant.

3. Ensure that all information is accurate and up to date.

4. Format the list of articles in a clear and organized manner, using a consistent style for each entry.

5. Include any additional information or notes that may be relevant or helpful for readers.

6. Double-check the accuracy and completeness of the list before publishing or submitting it.

*Prompt 2 (Complex):*

Act as an experienced researcher. Compile a list of ten recent articles on this question: Is an artificial pancreas or insulin pump effective in a second-trimester pregnant lady presenting with difficult-to-manage gestational diabetes?

Include the following information for each article:

1. Article title.

2. Author(s).

3. Journal name.

4. Date of publication.

5. Number of citations.

6. DOI.

7. Web link to the article.

8. PubMed link.

Instructions:

1. Begin by searching PubMed.

2. Review the search results and select ten recent articles that are relevant.

3. Ensure that all information is accurate and up to date.

4. Format the list of articles in a clear and organized manner, using a consistent style for each entry.

5. Include any additional information or notes that may be relevant or helpful for readers.

6. Double-check the accuracy and completeness of the list before publishing or submitting it.

*Prompt 3 (Basic):*

Act as an experienced researcher. Compile a list of ten recent articles on the exacerbation triggering factors in elderly asthmatic population.

Include the following information for each article:

1. Article title.

2. Author(s).

3. Journal name.

4. Date of publication.

5. Number of citations.

6. DOI.

7. Web link to the article.

8. PubMed link.

Instructions:

1. Begin by searching PubMed.

2. Review the search results and select ten recent articles that are relevant.

3. Ensure that all information is accurate and up to date.

4. Format the list of articles in a clear and organized manner, using a consistent style for each entry.

5. Include any additional information or notes that may be relevant or helpful for readers.

6. Double-check the accuracy and completeness of the list before publishing or submitting it.

*Prompt 4 (Complex):*

Act as an experienced researcher. Compile a list of ten recent articles on the best treatment regimen for poorly controlled asthma in the elderly with chronic rhinosinusitis and nasal polyps and congestive heart failure.

Include the following information for each article:

1. Article title.

2. Author(s).

3. Journal name.

4. Date of publication.

5. Number of citations.

6. DOI.

7. Web link to the article.

8. PubMed link.

Instructions:

1. Begin by searching PubMed.

2. Review the search results and select ten recent articles that are relevant.

3. Ensure that all information is accurate and up to date.

4. Format the list of articles in a clear and organized manner, using a consistent style for each entry.

5. Include any additional information or notes that may be relevant or helpful for readers.

6. Double-check the accuracy and completeness of the list before publishing or submitting it.

*Prompt 5 (Basic):*

Act as an experienced researcher. Compile a list of ten recent articles on the causes of septic shock in infants with severe combined immunodeficiency (SCID).

Include the following information for each article:

1. Article title.

2. Author(s).

3. Journal name.

4. Date of publication.

5. Number of citations.

6. DOI.

7. Web link to the article.

8. PubMed link.

Instructions:

1. Begin by searching PubMed.

2. Review the search results and select ten recent articles that are relevant.

3. Ensure that all information is accurate and up to date.

4. Format the list of articles in a clear and organized manner, using a consistent style for each entry.

5. Include any additional information or notes that may be relevant or helpful for readers.

6. Double-check the accuracy and completeness of the list before publishing or submitting it.

*Prompt 6 (Complex):*

Act as an experienced researcher. Compile a list of ten recent articles on this question: Is extracorporeal membrane oxygenation (ECMO) effective in pediatric patients with severe combined immunodeficiency (SCID), post bone marrow transplantation (BMT), admitted in PCIU with severe ARDS, graft-versus-host disease, septic shock, and multiorgan dysfunction syndrome?

Include the following information for each article:

1. Article title.

2. Author(s).

3. Journal name.

4. Date of publication.

5. Number of citations.

6. DOI.

7. Web link to the article.

8. PubMed link.

Instructions:

1. Begin by searching PubMed.

2. Review the search results and select ten recent articles that are relevant.

3. Ensure that all information is accurate and up to date.

4. Format the list of articles in a clear and organized manner, using a consistent style for each entry.

5. Include any additional information or notes that may be relevant or helpful for readers.

6. Double-check the accuracy and completeness of the list before publishing or submitting it.

*Prompt 7 (Basic):*

Act as an experienced researcher. Compile a list of ten recent articles on the different mechanisms of arthritis in patients with sickle cell disease.

Include the following information for each article:

1. Article title.

2. Author(s).

3. Journal name.

4. Date of publication.

5. Number of citations.

6. DOI.

7. Web link to the article.

8. PubMed link.

Instructions:

1. Begin by searching PubMed.

2. Review the search results and select ten recent articles that are relevant.

3. Ensure that all information is accurate and up to date.

4. Format the list of articles in a clear and organized manner, using a consistent style for each entry.

5. Include any additional information or notes that may be relevant or helpful for readers.

6. Double-check the accuracy and completeness of the list before publishing or submitting it.

*Prompt 8 (Complex):*

Act as an experienced researcher. Compile a list of ten recent articles on this case: A 17-year-old sickle cell disease patient has a history of recurrent episodes of Vaso occlusive crises and recurrent strokes, managed with monthly blood exchange transfusions for the last two years, presented with acute knee joint swelling. What are the possible pathological explanations?

Include the following information for each article:

1. Article title.

2. Author(s).

3. Journal name.

4. Date of publication.

5. Number of citations.

6. DOI.

7. Web link to the article.

8. PubMed link.

Instructions:

1. Begin by searching PubMed.

2. Review the search results and select ten recent articles that are relevant.

3. Ensure that all information is accurate and up to date.

4. Format the list of articles in a clear and organized manner, using a consistent style for each entry.

5. Include any additional information or notes that may be relevant or helpful for readers.

6. Double-check the accuracy and completeness of the list before publishing or submitting it.

*Prompt 9 (Basic):*

Act as an experienced researcher. Compile a list of ten recent articles on the causes of substance abuse in patients with a personality disorder.

Include the following information for each article:

1. Article title.

2. Author(s).

3. Journal name.

4. Date of publication.

5. Number of citations.

6. DOI.

7. Web link to the article.

8. PubMed link.

Instructions:

1. Begin by searching PubMed.

2. Review the search results and select ten recent articles that are relevant.

3. Ensure that all information is accurate and up to date.

4. Format the list of articles in a clear and organized manner, using a consistent style for each entry.

5. Include any additional information or notes that may be relevant or helpful for readers.

6. Double-check the accuracy and completeness of the list before publishing or submitting it.

*Prompt 10 (Complex):*

Act as an experienced researcher. Compile a list of ten recent articles on the possible mechanisms of depression and anxiety symptoms in a patient admitted over ingestion of herbs and dietary supplements.

Include the following information for each article:

1. Article title.

2. Author(s).

3. Journal name.

4. Date of publication.

5. Number of citations.

6. DOI.

7. Web link to the article.

8. PubMed link.

Instructions:

1. Begin by searching PubMed.

2. Review the search results and select ten recent articles that are relevant.

3. Ensure that all information is accurate and up to date.

4. Format the list of articles in a clear and organized manner, using a consistent style for each entry.

5. Include any additional information or notes that may be relevant or helpful for readers.

6. Double-check the accuracy and completeness of the list before publishing or submitting it.


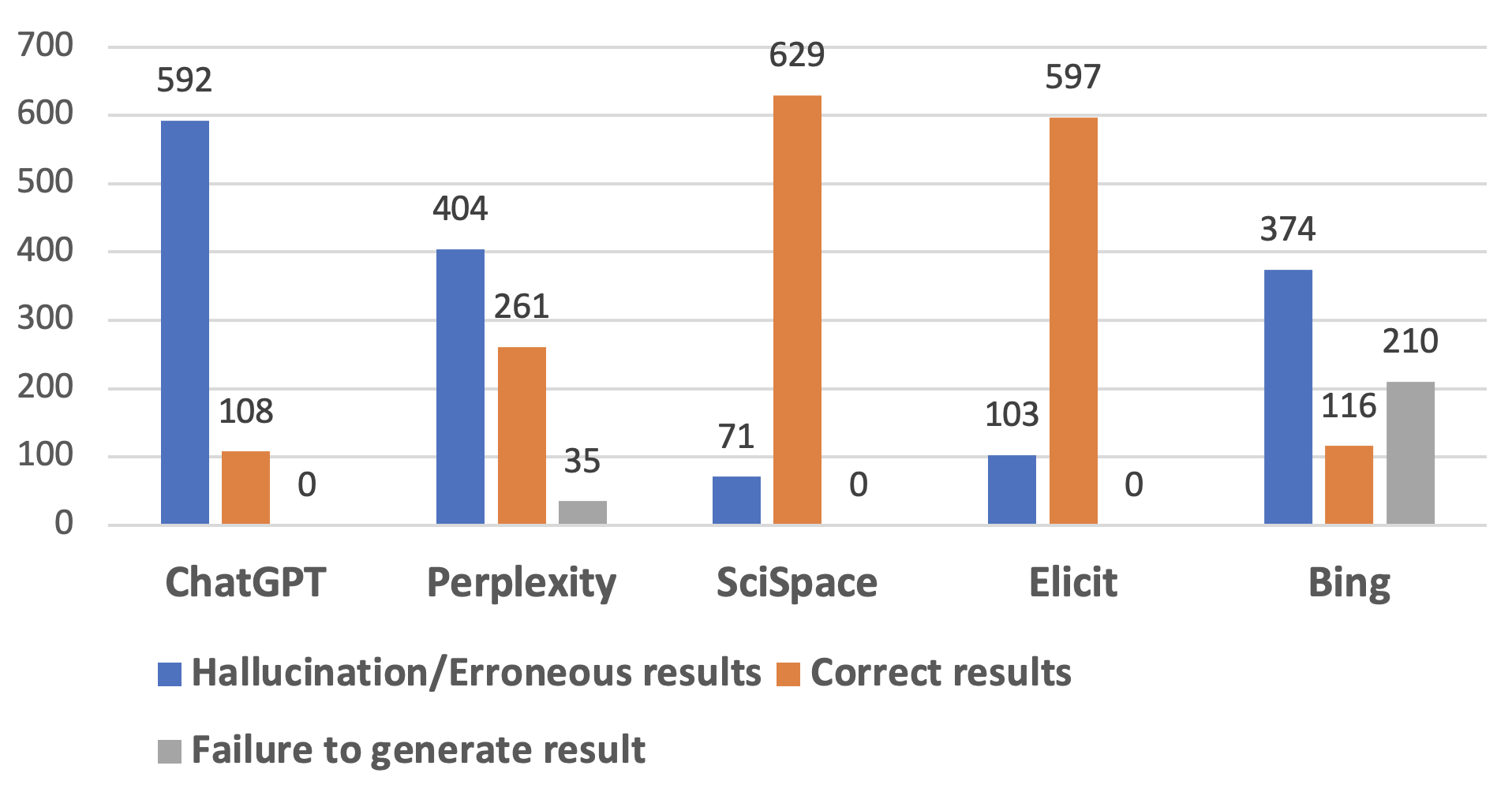


**Figure S1.** Individual AI chatbots’ reference results, including hallucination, correct, and failure of generation.
